# CCL20 mediates RANK/RANKL-induced epithelial-mesenchymal transition in endometrial cancer cells

**DOI:** 10.18632/oncotarget.8291

**Published:** 2016-03-23

**Authors:** Yao Liu, Jing Wang, Ting Ni, Lihua Wang, Yudong Wang, Xiao Sun

**Affiliations:** ^1^ Department of Gynecology, International Peace Maternity and Child Health Hospital, School of Medicine, Shanghai Jiao Tong University, Shanghai, China; ^2^ Laboratory for Gynecologic Oncology, International Peace Maternity and Child Health Hospital, School of Medicine, Shanghai Jiao Tong University, Shanghai, China

**Keywords:** CCL20, RANK, RANKL, epithelial-mesenchymal transition, endometrial cancer

## Abstract

RANK/RANKL facilitates migration/invasion via epithelial-mesenchymal transition (EMT) in certain malignant tumors. The relationship and mechanism between RANK/RANKL and EMT in endometrial cancer (EC) cells, however, remain unclear. In this study, we firstly showed that RANK/RANKL activation was correlated with EC staging and EMT markers in human EC tissue specimen. RANK/RANKL promoted migration/invasion and initiated EMT of EC cell lines. Then, protein chip analysis and enzyme-linked immunosorbent assay (ELISA) revealed that the expression and secretion of chemokine ligand 20 (CCL20) was dramatically enhanced in RANKL-treated RANK over-expressed EC cells. Moreover, the higher level of CCL20 in both serum and tumor tissue was detected in orthotopic transplantation mouse models. Finally, we confirmed that CCL20 contributed to invasion and EMT of RANK over-expressed EC cells. In summary, all data supported the hypothesis that RANK/RANKL elevated the expression and secretion of CCL20 in EC cells, which promoted cancer progression through EMT.

## INTRODUCTION

Endometrial cancer (EC) remains one of the most common gynecological malignancies worldwide. In United States, it ranks fourth place, with about 54,870 incident cases and 10,170 deaths in 2015 [1]. Despite recent medical progresses in surgical treatment for early-stage EC, therapy for advanced EC is still less effective [2, 3]. As we know, metastasis is the primary cause of poor prognosis for EC patients [4]. Therefore, illuminating the mechanisms that facilitate metastasis is of great concern. Based on present studies, cytokines and chemokines existing in the tumor microenvironment were supposed to promote tumor initiation, metastasis and progression [5, 6].

Receptor activator of nuclear factor-κB (RANK) and its ligand RANKL were shown to regulate osteoclast differentiation, bone remodeling and the formation of lymph node. Recent studies reported that RANKL accelerated invasion and metastasis in RANK-expressed cancer cells [7–9]. Also, Palafox et al. [10] found that RANK/RANKL induced EMT in human mammary epithelial cells. Our previous study confirmed that the activation of RANK/RANKL could obviously promote EC metastasis in animal orthotopic transplantation mode [11, 12]. However, it remains elusive whether RANK/RANKL could induce EMT in EC cells.

Some chemokines derived from tumor-associated leukocytes or tumor cells acted as growth factors for cancer cells. They could contribute to tumor metastasis by accelerating angiogenesis, attracting endothelial cells or regulating the motility ability of cancer cells. Among them, CCL20 had proved to be related to invasion and metastasis in some types of cancer [13]. CCL20 was identified in the liver for the first time [14]. It was initially named as liver and activation regulated chemokine (LARC) and alternatively called Exodus-1 or macrophage inflammatory protein-3a (MIP-3a). CCL20 was strongly chemotactic for lymphocytes as well as weakly attracted neutrophils [15]. Accumulating evidence showed that CCL20 was correlated with tumor formation, metastasis or progression in many malignant neoplasms, such as colorectal and breast cancer [16, 17]. In addition, several chemokines including CCL20 had been reported to induce EMT in various tumors [18, 19, 20]. EMT played a key role in tumor progression, which leaded to a more invasive and metastatic phenotype in many human cancers, involving EC [21]. However, the relationship between CCL20 and EMT in EC cells is still unknown.

To clarify the mechanism of RANK/RANKL-induced EMT in EC, we built RANK over-expressed cell lines and animal models. Protein chip analysis and ELISA suggested that the expression and secretion of CCL20 was dramatically enhanced in RANKL-treated RANK over-expressed EC cells. Furthermore, CCL20 could facilitate migration/invasion and promote cancer progression through EMT. This work firstly illustrated mechanisms by which RANK over-expressed EC cells became invasive and metastatic. In addition, it supplied a potential therapeutic target for EC.

## RESULTS

### RANK/RANKL activation correlates with EC staging and EMT markers in human EC tissue specimens

**Figure 1:**
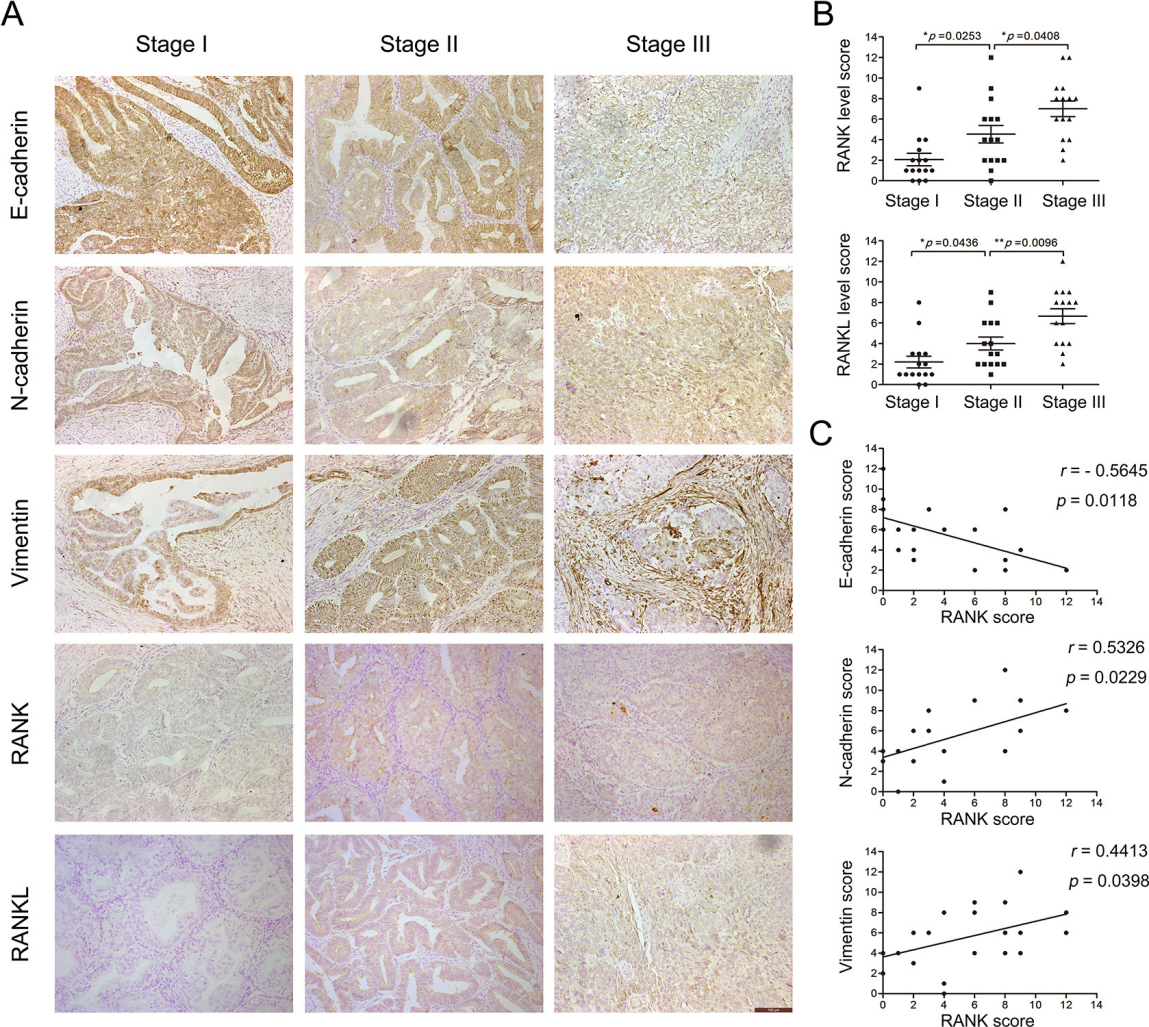
Expression of RANK/RANKL and EMT markers in human EC tissue specimens (**A**) IHC analysis of RANK, RANKL, E-cadherin, N-cadherin and Vimentin in EC (stage I, II, III). Amplification: 200×. Bar = 100 μm. (**B**) Semi-quantitative analysis of IHC staining determined the levels of RANK and RANKL in each stage of EC. (**C**) Expression correlations between RANK and E-cadherin, N-cadherin and Vimentin, respectively. Spearman's correlation coefficient test was used to statistical analysis.

To clarify the effect of RANK/RANKL on the progression and EMT in EC, we used immunohistochemistry to detect levels of RANK, RANKL and EMT markers in human EC tissue specimens with different stage (I, II, III). The data showed that RANK/RANKL expression was significantly elevated in EC tissue of higher stage (Figure 1A, 1B). Moreover, RANK level was positively connected with N-cadherin (*p* = 0.0229) and Vimentin (*p* = 0.0398), but negatively with E-cadherin (*p* = 0.0118) (Figure 1A, 1C). This indicated that RANK/RANKL activation was related to EMT in EC.

### Overexpression of RANK contributes to migration and invasion of EC cell lines treated by RANKL

**Figure 2:**
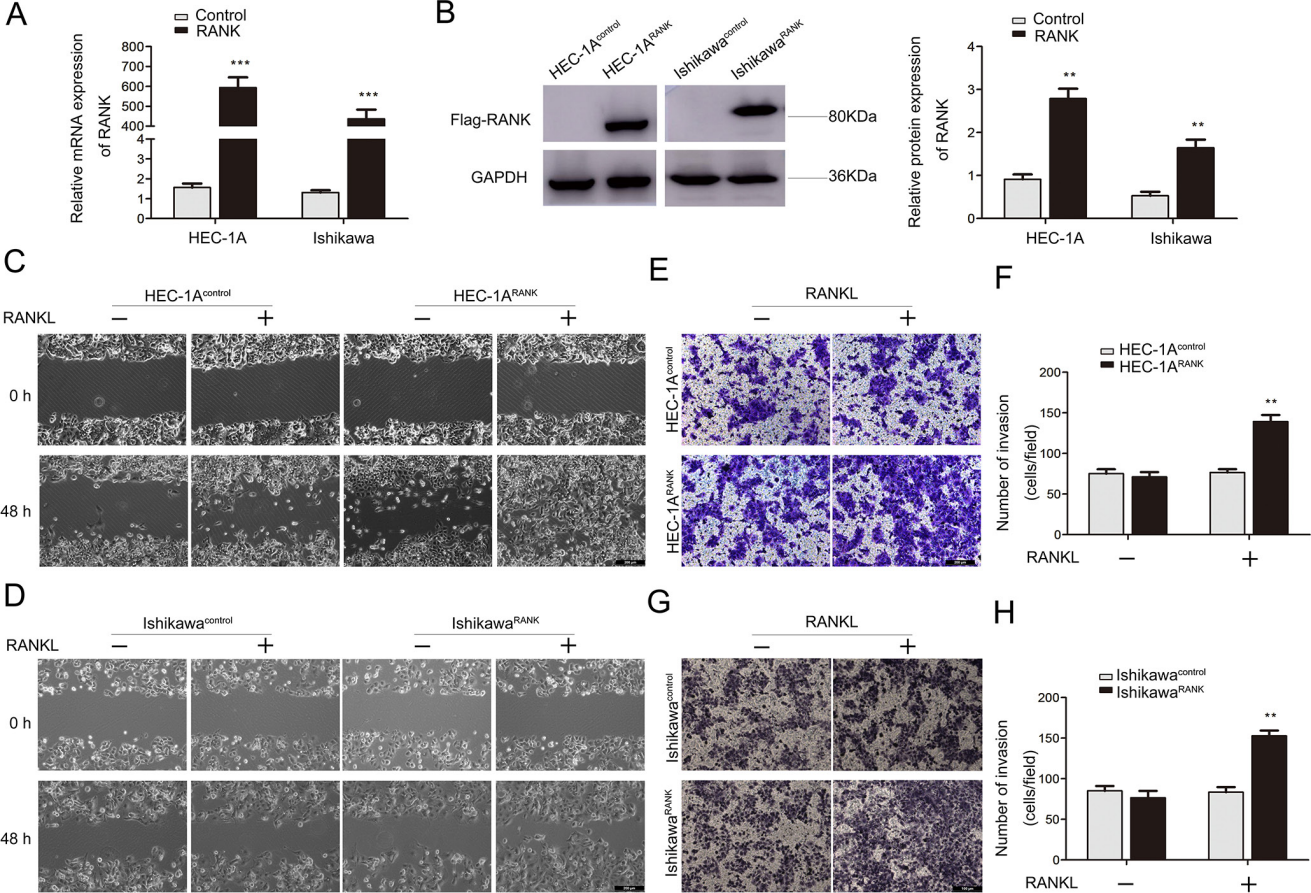
RANK/RANKL accelerates migration and invasion of EC cells (**A**, **B**) Overexpression of RANK in HEC-1A and Ishikawa cells revealed by qRT-PCR and western blotting, and the latter was further quantified by densitometry of three times. GAPDH was included as an internal control. **P < 0.01, ***P < 0.001, *n* = 3. (**C**, **D**) Wound-healing migration assays for HEC-1A and Ishikawa cells. The healing of wounds was imaged at 0 h and 48 h, showing that overexpression of RANK enhanced migration. The images were obtained at 100× magnification. Bar = 200 μm. (**E**, **G**) Transwell invasion assays. EC cells were grown in upper chamber for 48 h. Invaded cells were fixed, stained and imaged using an inverted microscope at 100 × magnification. Bar = 200 μm. (**F**, **H**) Graphs indicated the number of invasive cells for each group. **P < 0.01, *n* = 3.

RANK/RANKL was recently shown to promote invasion of cancer cells, but the underlying molecular mechanism remained to be determined. Here, the role of RANK/RANKL in EC cell lines was explored. In order to verify whether RANK/RANKL stimulated EC cells progression, the over-expression plasmid targeting RANK, pIRES2–3FLAG-EGFP-RANK, was inserted in HEC-1A and Ishikawa cells via transient transfection, which exhibited a constitutively active function of RANK receptor. Cells transfected with an empty plasmid, pIRES2–3FLAG-EGFP-CON236, served as the control. These cell lines were named HEC-1A^RANK^ or HEC-1A^Control^ and Ishikawa^RANK^ or Ishikawa^Control^. Efficient transfection was detected before performing cellular assays (Figure 2A, 2B).

In wound-healing migration assay, the wound area was monitored at 48 h after establishing the wound. The wound closure was reduced obviously in RANKL-treated HEC-1A^RANK^ and Ishikawa^RANK^ cells when compared with control groups (Figure 2C, 2D).

To further investigate the invasion ability of EC cells, we carried out transwell invasion assay. The average number of cells invaded into the lower chamber was counted under inverted microscope from 5 fields. Comparing to control groups, the invaded number of RANKL-treated RANK over-expressed EC cells was significantly elevated (Figure 2E–2H).

These results collectively demonstrated that the migration and invasion capabilities of EC cells were significantly promoted by RANK/RANKL.

### RANK/RANKL initiates EMT in EC cells

**Figure 3:**
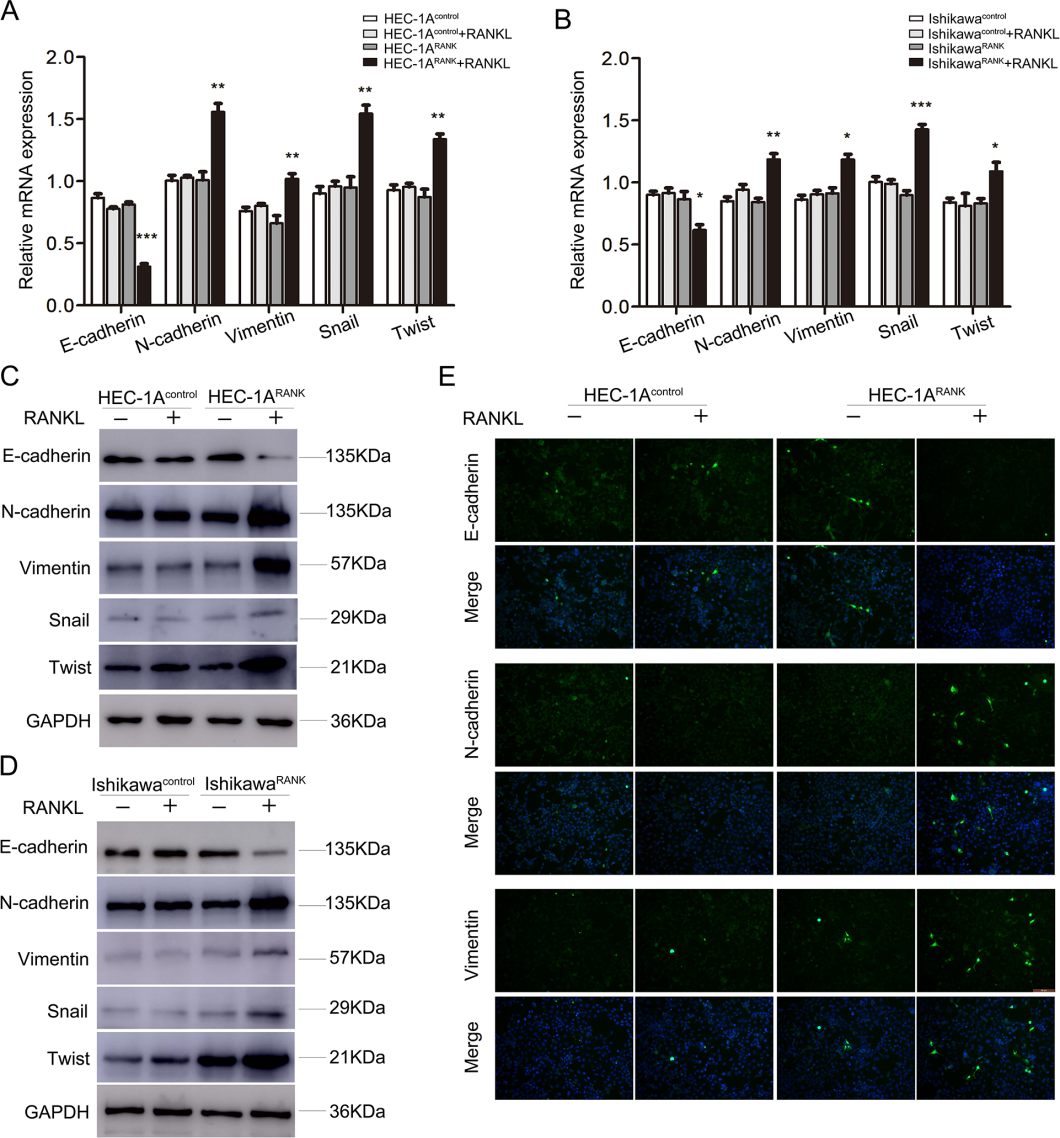
RANK/RANKL induces EMT in EC cells (**A**, **B**) HEC-1A and Ishikawa cells were transfected with an empty plasmid or over-expression plasmid targeting RANK. After 48 h stimulation of RANKL, the mRNA levels of E-cadherin, N-cadherin, Vimentin, Snail and Twist were analyzed by qRT-PCR. *P < 0.05, **P < 0.01, ***P < 0.001, *n* = 3. (**C**, **D**) HEC-1A and Ishikawa cells were transfected with an empty plasmid or over-expression plasmid targeting RANK. After 48 h stimulation of RANKL, the protein expression of E-cadherin, N-cadherin, Vimentin, Snail and Twist were detected by western blotting. (**E**) Immunofluorescence staining showed the expression of E-cadherin, N-cadherin and Vimentin in RANKL-treated HEC-1A^Control^ and HEC-1A^RANK^ cells. The images were obtained under fluorescence microscopy. Bar = 50 μm.

EMT was closely correlated with tumor metastasis and progression. To explore the relationship between RANK/RANKL and EMT in EC cells, we microscopically examined the change in morphology of RANK-overexpressed EC cells following stimulation with RANKL. After 48 h of treatment, HEC-1A^RANK^ and Ishikawa^RANK^ cells were morphologically transformed toward mesenchymal fibroblastic spindle shape compared with control groups, suggesting a phenotypic transition from epithelial to mesenchymal (data not shown). Then, to test whether this morphological change represented EMT, we detected the expression of several EMT markers from multiple levels. Quantitative real-time PCR analysis indicated that the mRNA level of E-cadherin was decreased, whereas levels of N-cadherin, Vimentin, Snail and Twist were increased in RANKL-treated HEC-1A^RANK^ and Ishikawa^RANK^ cells (Figure 3A, 3B). Western blotting and immunofluorescence staining analysis also showed that the overexpression of RANK led to down-regulation of E-cadherin, but up-regulation of N-cadherin, Vimentin, Snail and Twist in EC cells under RANKL treatment (Figure 3C–3E).

Taken together, our data suggested that RANK/RANKL could induce EMT in EC cells.

### RANK/RANKL promotes the expression and secretion of CCL20 *in vitro* and *in vivo*

**Figure 4:**
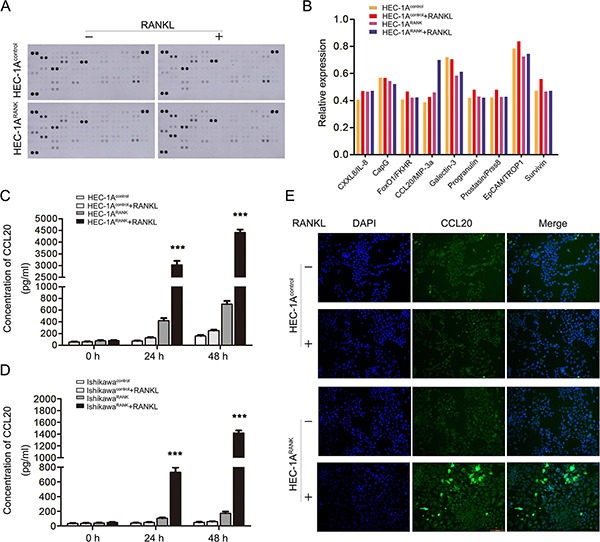
RANK/RANKL increases the expression and secretion level of CCL20 in EC cells (**A**) The Human XL Oncology Array detected multiple analytes in cell culture lysates. The lysates were gained from HEC-1A^Control^ and HEC-1A^RANK^ cells under the treatment of RANKL for 48 h. (**B**) The graph summarized the relative signal intensity of indicated molecules. Among them, CCL20 varied most significantly. (**C**, **D**) The cell supernatant was collected from HEC-1A/Ishikawa^Control^ cells and HEC-1A/Ishikawa^RANK^ cells treated by RANKL for 48 h. The serum concentration of CCL20 was measured with ELISA kit. ***P < 0.001, *n* = 3. (**E**) Immunofluorescence staining showed the expression of CCL20 in HEC-1A^Control^ and HEC-1A^RANK^ cells after 48 h stimulation of RANKL. The images were obtained by fluorescence microscopy. Bar = 100 μm.

To identify the novel mediators of EMT in EC cells, RANKL-treated HEC-1A^RANK^ and HEC-1A^Control^ cells were analyzed using Human XL Oncology Array Kit from RandD Systems. The expression of CCL20 was elevated in RANKL-treated HEC-1A^RANK^ cell revealed by protein chip analysis (Figure 4A, 4B). Then, ELISA was performed to detect the serum level of CCL20 in different treatment groups. The serum concentration of CCL20 in RANKL-treated HEC-1A^RANK^ and Ishikawa^RANK^ cells was prominently higher than that in control groups, and this effect was time-dependent (Figure 4C, 4D). In addition, immunofluorescence staining suggested that CCL20 expression was enhanced in RANKL-treated HEC-1A^RANK^ cell (Figure 4E).

**Figure 5:**
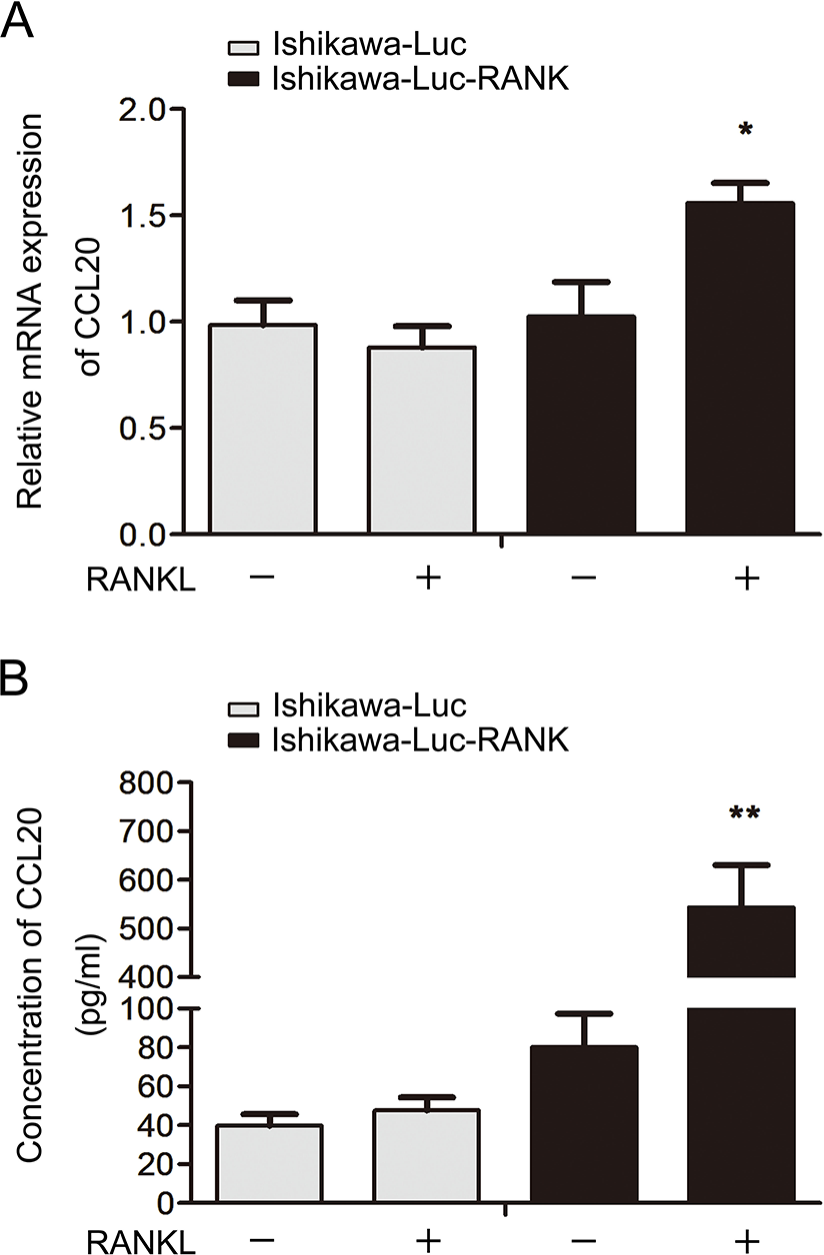
Expression and secretion level of CCL20 elevates in nude mice orthotopic transplantation modes (**A**) Tumor tissues were removed from tumor-bearing mouse after four weeks’ injection of RANKL. The mRNA expression of CCL20 was detected by qRT-PCR. *P < 0.05, *n* = 3. (**B**) Serum from each group was collected when the mice were sacrificed immediately. The serum level of CCL20 was measured using the ELISA kit. **P < 0.01, *n* = 3.

So as to further evaluate the connection between CCL20 and RANK/RANKL in EC, we built animal transplantation models, detecting the expression and secretion of CCL20 *in vivo*. We gained Ishikawa-Luc/Ishikawa-Luc-Rank light-emitting EC cells to establish orthotopic xenograft nude model. After four weeks’ injection of RANKL, the mice were sacrificed. Tumor tissues were then removed from animals, and grinded to obtain mRNA. qRT-PCR analysis showed that the mRNA level of CCL20 was higher in RANKL-treated group (Figure 5A). Meanwhile, the serum was collected to measure the concentration of CCL20 via ELISA. We discovered that the secretion of CCL20 was also notably increased in RANKL-treated group (Figure 5B).

### CCL20 facilitates invasion and EMT of RANK over-expressed EC cells

**Figure 6:**
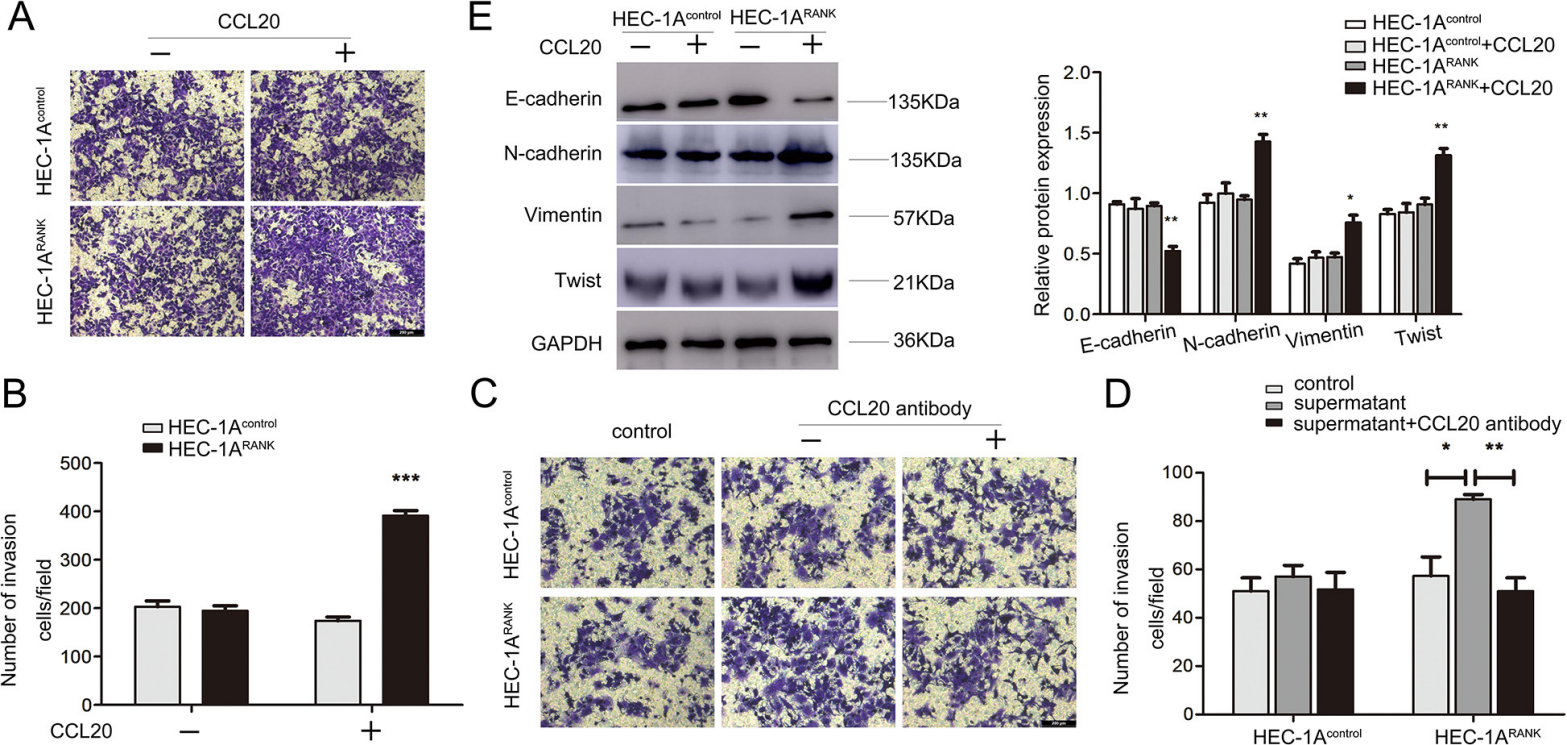
CCL20 facilitates invasion and EMT of RANK over-expressed EC cells (**A**) HEC-1A^Control^ and HEC-1A^RANK^ cells were grown in upper chamber without or with the co-culture of exogenous CCL20 (50 ng/ml) for 48 h. Invaded cells were fixed, stained and imaged under an inverted microscope at 100× magnification. Bar = 200 μm. (**B**) Quantitative analysis of invaded cell number from five random fields. ***P < 0.001, *n* = 3. (**C**) HEC-1A^Control^ and HEC-1A^RANK^ cells were grown in upper chamber. Then, added the conditional medium of RANKL-treated HEC-1A^RANK^ cell without or with neutralizing antibody against CCL20 to the lower chambers. After 48 h, representative images were obtained at 200× magnification. Bar = 200 μm. (**D**) Graphs indicated the number of invaded cells for each group. *P < 0.05, **P < 0.01, *n* = 3. (**E**) HEC-1A^Control^ and HEC-1A^RANK^ cells were treated by CCL20 for 48 h. The protein expression of E-cadherin, N-cadherin, Vimentin and Twist were measured. Quantitative analysis of the results was further shown. *P < 0.05, **P < 0.01, *n* = 3.

To better understand the effect of CCL20 on metastasis of EC cells, chamber co-culture assays were carried out. As shown in Figure 6A and 6B, the number of invaded HEC-1A^RANK^ cells was more than HEC-1A^Control^ cells with the co-culture of exogenous CCL20. Besides, when the conditional medium of RANKL-treated HEC-1A^RANK^ cells that included massive endogenous CCL20 was added to lower chambers, HEC-1A^RANK^ cells exhibited stronger invasion ability. Addition of neutralizing antibody against CCL20, however, prominently blocked the capability of CCL20 to promote invasion (Figure 6C, 6D).

In order to clarify the relation between CCL20 and EMT in EC cells, HEC-1A^Control^ and HEC-1A^RANK^ cells were treated with CCL20 for 48 h, and then the levels of EMT markers were measured. Western blotting analysis showed that the expression of E-cadherin was decreased, whereas N-cadherin, Vimentin and Twist were increased in CCL20-stimulated HEC-1A^RANK^ cells (Figure 6E). Collectively, these data indicated that CCL20 could induce EMT in RANK over-expressed EC cells.

## DISCUSSION

Tumor metastasis and progression are complex and multistep processes, which make them the leading cause of recurrence and death in cancer patients. Consequently, elucidating molecular mechanisms that promote metastasis is indispensable to seek new therapeutic strategies and improve therapeutic effects of patients with EC [22].

RANK/RANKL is needful for normal physiological processes such as immune responses and bone remodeling [23]. Also, it facilitates invasion and metastasis of human cancers [24]. Our previous study showed that RANK/RANKL expression was higher in EC tissue, and could dramatically promote EC metastasis [11, 12]. Here, we concluded that over-expression of RANK contributed to migration and invasion of EC cell lines under the stimulation of RANKL.

EMT correlates with tumor metastasis and progression. A notable EMT feature is the loss of E-cadherin, which enables a cell to dissolve cell-cell adhesion and break away from its neighbors. Instead, mesenchymal markers like N-cadherin and fibronectin, are increased to facilitate invasion and migration [25, 26]. Recent studies reported that EMT heralded aggressive tumor features and poor prognosis in EC [27, 28]. In this paper, we discovered that there was a morphological transformation in RANKL-treated HEC-1A^RANK^ and Ishikawa^RANK^ cells. A down-regulation of E-cadherin and up-regulation of N-cadherin, Vimentin, Snail and Twist were also detected. Thus, we concluded that RANK/RANKL could induce EMT in EC cells.

**Figure 7:**
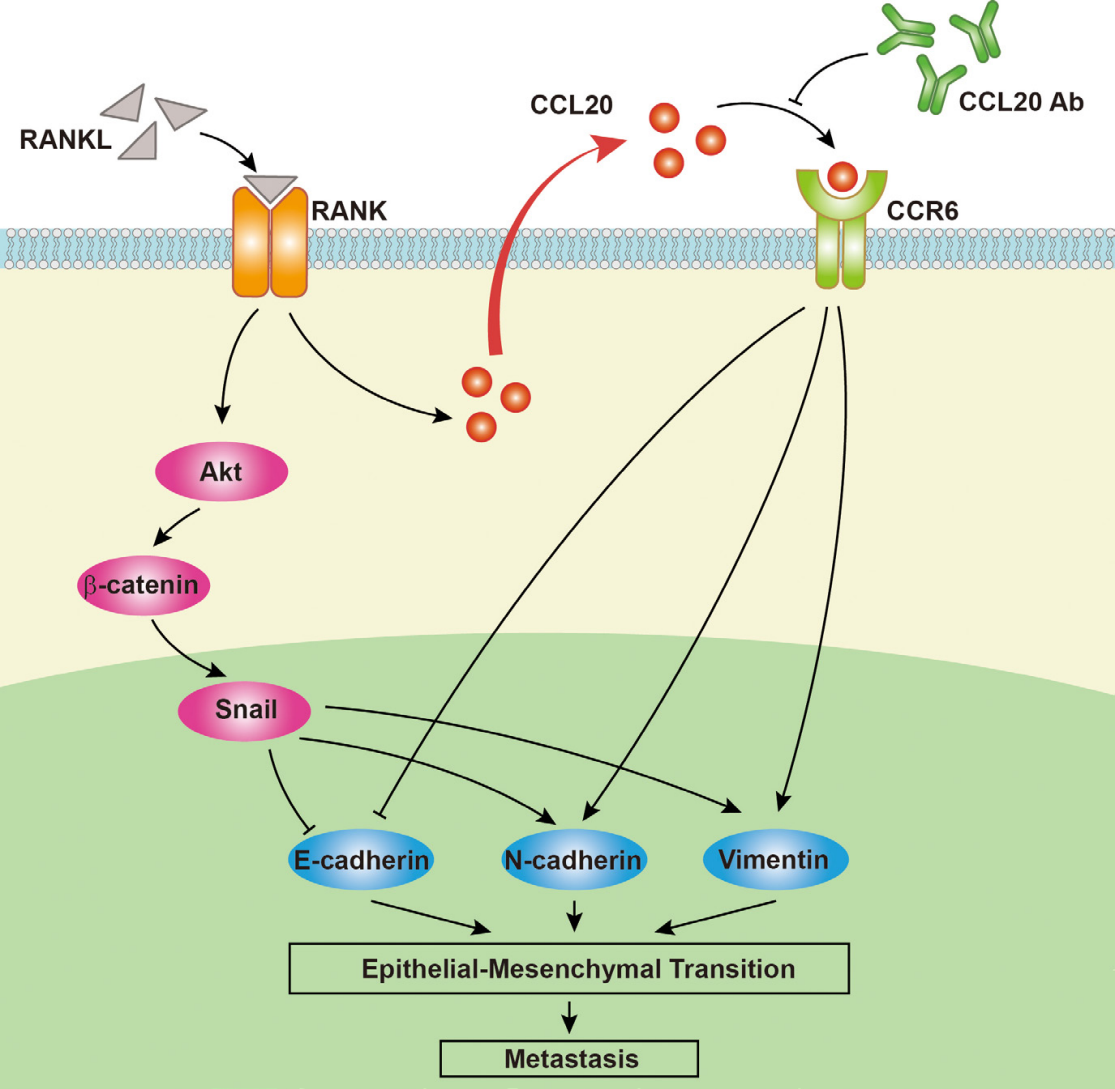
A proposed model for the role of CCL20 in RANK/RANKL-induced EMT in EC cells RANK/RANKL up-regulates the expression and secretion of CCL20 in EC cells. CCL20 could bind to its solely receptor CCR6. Then, this interplay contributes to the migration/invasion and promotes EC progression through EMT. Furthermore, neutralizing antibody against CCL20 could refrain from this effect.

In reality, cancer metastasis is not due to a single genetic variation, but requires the participation of multiple factors, both within cells and in the tumor microenvironment. Consequently, the important role of chemokines is emerging. Chemokines play an essential part in various biologic events, such as regulating the migration of white blood cells, tissue architecture, Th1/Th2 development and leukocyte homeostasis [29]. Recently, there were several publications supporting that chemokines were critical in tumor metastasis [30–36] and inhibition of them reduced the advancement of metastasis *in vivo* [37]. Acting in both a paracrine fashion to regulate the activity of neighboring cells and an autocrine manner, chemokines perform various functions. Abnormal secretion of chemokines by tumor-associated macrophages has been involved in tumor cell survival, angiogenesis, migration/invasion and regulation of the immune system interacting with the tumor [38, 39]. Furthermore, chemokines released by the tumor cells themselves provide cancer cells with a ready-made route towards the adjacent tissues or blood stream. Several chemokines including CCL20 have been reported to induce EMT in various tumors. Biswas et al. [18] showed that CXCL13-CXCR5 initiated EMT procession of breast cancer cells. Li et al. [19] indicated that SDF-1/CXCR4 promoted metastasis and EMT *in vitro* via non-canonical hedgehog pathway in pancreatic cancer. Matsushita et al. [20] found that CXCL16 contributed to liver metastasis of colorectal carcinoma by inducing EMT. Here, we discovered that the expression and secretion of CCL20 was prominently increased in RANKL-treated RANK over-expressed EC cells *in vitro* and *in vivo*. In addition, CCL20 accelerated invasion and induced EMT of RANK over-expressed EC cells, and neutralizing antibody against CCL20 could suppress this effect. Therefore, our study presented another model of chemokine-mediated EMT process, in which CCL20 may play a crucial role in EC metastasis and progression (Figure 7). Interestingly, it was true that similar chemokines networks were activated in different tumors. This indicated that underlying damage of tumor immune-surveillance led to subsequent extensive metastasis. Our data supported the hypothesis that CCL20 was elevated in RANKL-treated RANK over-expressed EC cells, and then contributed to immune suppression-mediated cancer cells metastasis via EMT.

In conclusion, we summarized that RANK/RANKL up-regulated the expression and secretion of CCL20 in EC cells, which accelerated migration/invasion and promoted cancer progression through EMT. Consequently, CCL20 may represent a promising molecular marker for preventing EC metastasis and progression.

## MATERIALS AND METHODS

### Clinical samples

45 samples of EC without hormone therapy, radiotherapy, or chemotherapy before surgery were obtained from the International Peace Maternity and Child Health Hospital during 2013 and 2014. The tumor stages and histological grades were established in line with the criteria of Federation International of Gynecology and Obstetrics (FIGO) 2009 staging system [40].

### Reagents and antibodies

Recombinant human RANK ligand and recombinant human MIP-3α/CCL20 were purchased from PeproTech (Rocky Hill, NJ, USA). Rabbit monoclonal antibodies (mAb) against E-cadherin and Vimentin were obtained from Cell Signaling Technology (Danvers, MA, USA). Rabbit polyclonal antibody against N-cadherin, Snail and Twist was from Abcam. Mouse mAb against FLAG was purchased from Cell Signaling Technology. Mouse mAb against RANK and goat polyclonal antibody against RANKL were from SANTA CRUZ (Delaware Avenue, CA, USA). Goat polyclonal antibody CCL20, Human XL Oncology Array Kit and Quantikine ELISA Kit were all purchased from R & D Systems (USA).

### Immunohistochemistry (IHC) and assessments

The paraffin-embedded EC tissues were sliced into 4 μm section, dewaxed with xylene and rehydrated with graded alcohol. Ethylene diamine tetraacetic acid was used for antigen retrieval, and 3% hydrogen peroxide was used for blocking endogenous peroxidase activity. Then, sections were incubated with primary antibodies against RANK (1:200), RANKL (1:200), E-cadherin (1:400), N-cadherin (1:250) and Vimentin (1:100) at 4°C overnight and with secondary antibodies for 30 minutes at room temperature. Two pathologists blinded to clinical pathological parameters assessed immune-reactivity scores (IRSs) based on Remmele and Stegner [41]. Staining intensity was scored as negative (0), weak (1), moderate (2), or strong (3). The percentage of positive cells was scored as 0 (0–5%), 1 (6–25%), 2 (26–50%), 3 (51–75%), and 4 (76–100%). Then, multiplying these two scores calculates IRS.

### Cell culture

Human EC cell lines (HEC-1A and Ishikawa cell) were obtained from the American Type Culture Collection (ATCC, Manassas, vA, USA). HEC-1A and Ishikawa cells were cultured in Dulbecco's modified Eagle medium (DMEM)/F12 (Gibco, Auckland, New Zealand) supplemented with 10% fetal bovine serum (Gibco, Carlsbad, CA, USA), 100 μg/ml penicillin and 100 U/ml streptomycin (Gibco). Cells were cultured in a humidified atmosphere of 5% CO_2_ at 37°C.

### Transient transfection

The over-expression plasmid targeting RANK, pIRES2-3FLAG-EGFP-RANK, and the empty plasmid, pIRES2-3FLAG-EGFP-CON236, were purchased from GeneChem Biotech (Shanghai, China). Transient transfection was carried out with 70% confluent EC cells and Lipofectamine 2000 reagents (Invitrogen). Then, both HEC-1A/Ishikawa^Control^ cells and HEC-1A/Ishikawa^RANK^ cells were treated by 1 ug/ml RANKL (PeproTech, USA) for 48 h.

### RNA isolation and quantitative real-time PCR (qRT-PCR) assays

Total RNA was isolated using Trizol reagent (Invitrogen, Shanghai, China) and the quality was assessed using spectrophotometer (Pharmacia Biotech RNA/DNA calculator). The first-strand cDNA was inversely transcribed from total RNA (1 μg) using a reverse transcription kit (TaKaRa, Dalian, China). Then, qRT-PCR was performed to analyze cDNA using SYBR Premix Ex Taq (Takara Biomedical) on an ABI Prism 700 thermal cycler (Applied Biosystems, Foster City, CA, USA). The qRT-PCR conditions for glyceraldehyde-3-phosphate dehydrogenase (GAPDH), E-cadherin, N-cadherin, Vimentin, Snail and Twist were 95°C 2 min, followed by 40 cycles of 95°C 10s, 60°C 30s. Primer sequences were as follows: GAPDH, forward primer 5′-ACAACTTTGGTATCGTGGAAGG-3′ and reverse primer 5′-GCCATCACGCCACAGTTTC-3′; E-cadherin, forward primer 5′-CGAGAGCTACACGTTCACGG-3′ and reverse primer 5′-GGGTGTCGAGGGAAAAATAGG-3′; N-cadherin, forward primer 5′-TGCGGTACAGTGTA ACTGGG-3′ and reverse primer 5′-GAAACCGGGCTA TCTGCTCG-3′; Vimentin, forward primer 5′-TGCCGTT GAAGCTGCTAACTA-3′ and reverse primer 5′-CCAGAG GGAGTGAATCCAGATTA-3′, Snail, forward primer 5′-ACTGCAACAAGGAATACCTCAG-3′ and reverse primer 5′-GCACTGGTACTTCTTGACATCTG-3′; Twist, forward primer 5′-ATTCAAAGAAACAGGGCGTGG-3′ and reverse primer 5′-CCTTTCAGTGGCTGATTGGC-3′. Values of vertical axis represent 2^(−ΔCt)^, and ΔCt is the discrepancy between target genes Ct and GAPDH Ct.

### Western blotting analysis

Treated cells were lysed in RIPA buffer containing protease inhibitor phenylmethanesulfonyl fluoride (Beyotime, Nanjing, China). The BCA Protein Assay kit (Beyotime, Nanjing, China) was used to determine protein concentrations. Equal amounts of extracts (50 μg of protein) were fractionated on each lane of a polyacrylamide-sodium dodecyl sulfate (SDS) gels and transferred to polyvinylidenefluoride (PVDF) membrane (Millipore, Billerica, MA). Then membranes were blocked with 5% skimmed milk for 2 h and incubated with antibodies against E-cadherin (1:1000), N-cadherin (1:1000), Vimentin (1:1000), Snail (1:1000), Twist (1:1000), GAPDH (1:1000) and FLAG (1:1000) at 4°C overnight. Subsequently, the membranes were incubated with secondary antibodies for 1 h at room temperature. The target proteins were visualized by adding ECL luminous agent using the image-forming system of Amersham Imager 600. GAPDH antibody was used as the internal standard.

### Wound healing assays

The transfected cells were seeded in a 60-mm plate. A 10 μl pipette tip was applied to wound the cell monolayer. After 48 h treatment of RANKL (1 μg/ml), cells were washed with PBS. The images were obtained under an inverted microscope.

### Transwell invasion assays

The upper transwell chambers (8-μm pore) were coated with 100 μl of Matrigel at a dilution of 1:6 (BD Biosciences, San Jose CA, USA). A total of 1 × 10^5^-transfected cells were seeded into the top chamber of a 24-well polycarbonate transwell filter (Corning Incorporated, Glendale, AZ, USA). Then, cells were treated with RANKL (1 μg/ml) for 48 h. The numbers of crystal violet-stained cells in five random fields were counted using an inverted microscope.

### Protein chip analysis

Lysates from treated human EC cells were extracted with RIPA buffer containing protease inhibitor phenylmethanesulfonyl fluoride (Beyotime, Nanjing, China). Then, 200 μg of cell lysate was run on each array of the Human XL Oncology Array Kit from RandD Systems (Cat. ARY026, USA). The follow-up steps were carried out in accordance with manufacturer's instructions. Array images were collected and analyzed using the Amersham Imager 600 Imaging System.

### Enzyme-linked immunosorbent assay (ELISA)

The ELISA kit is an *in vitro* enzyme-linked immunosorbent assay for quantitative measurement. The supernatants of treated cells were stored at −80°C until assayed for CCL20 with Quantikine ELISA Kit from R & D Systems (Cat. DM3A00, USA). Standards and treated cells samples were both diluted in Calibrator Diluent RD6–21 obtained from the ELISA Kit. The optical density of each well was determined within 30 minutes via a microplate reader setting to 450 nm.

### Immunofluorescence assay

HEC-1A cells were grown on coverslips. Transient transfection was performed when cells grew to 70% confluent. Both HEC-1A^Control^ cells and HEC-1A^RANK^ cells were treated by RANKL (1 ug/ml) for 48 h. We fixed the cells with 4% paraformaldehyde for 15 min and blocked them with 0.5% (V/V) Triton X-100 in PBS for 30 min at room temperature. Then cells were incubated with primary antibodies against CCL20 (1:100), E-cadherin (1:200), N-cadherin (1:100) and Vimentin (1:100) at 4°C overnight. Finally, cells were incubated with secondary antibody coupled with fluorophores. Images were obtained by fluorescence microscopy.

### *In vivo* experiments

We gained Ishikawa-Luc/Ishikawa-Luc-Rank light-emitting EC cells to build orthotopic xenograft nude model. The establishment process of animal model was performed in terms of our previously study [12]. After four weeks’ injection of RANKL, the nude mice were sacrificed. Then, tumor tissues were removed from animals, and grinded to obtain mRNA. qRT-PCR was used to analyze the mRNA level of CCL20. Meanwhile, the serum was collected to measure the concentration of CCL20 with ELISA kit.

### Statistical analysis

We performed all statistical analyses with SPSS software, version 17.0 (SPSS, Inc., Chicago, IL, USA). The data was analyzed using an unpaired Student's *t*-test or one-way ANOVA. The correlation detection was performed by Spearman's correlation coefficient test. Each experiment was carried out in triplicate. *P*-values of < 0.05 were considered statistically significant.
